# Peripheral blood markers predictive of outcome and immune-related adverse events in advanced non-small cell lung cancer treated with PD-1 inhibitors

**DOI:** 10.1007/s00262-020-02585-w

**Published:** 2020-04-29

**Authors:** Lihong Peng, Yong Wang, Fen Liu, Xiaotong Qiu, Xinwei Zhang, Chen Fang, Xiaoyin Qian, Yong Li

**Affiliations:** 1 Department of Medical Oncology, First Affiliated Hospital of Nanchang University, 17 Yongwai Zheng Road, Nanchang, 330000 China; 2 Department of Medical Oncology, Affiliated Ganzhou Hospital of Nanchang University (Ganzhou People’s Hospital), 18 Meiguan Road, Ganzhou, 341000 China; 3 Critical Care Medicine, First Affiliated Hospital of Nanchang University, 17 Yongwai Zheng Road, Nanchang, 330000 China

**Keywords:** Lung cancer, Immunotherapy, Immune-related adverse events, Neutrophil-to-lymphocyte ratio, Lactate dehydrogenase, Prognostic nutrition index

## Abstract

**Background:**

Selected patients with advanced non-small cell lung cancer (NSCLC) benefit from immunotherapy, especially immune checkpoint inhibitors such as PD-1 (programmed cell death protein 1) inhibitor. Peripheral blood biomarkers would be most convenient to predict treatment outcome and immune-related adverse events (irAEs) in candidate patients. This study explored associations between inflammation-related peripheral blood markers and onset of irAEs and outcome in patients with advanced NSCLC receiving PD-1 inhibitors.

**Methods:**

A retrospective analysis was conducted of 102 patients with advanced NSCLC receiving PD-1 inhibitors from January 2017 to May 2019. Cox regression models were employed to assess the prognostic effect of low/high neutrophil/lymphocyte ratio (NLR), lactate dehydrogenase (LDH), and prognostic nutrition index (PNI) on overall survival (OS) and progression-free survival (PFS). Logistic regression models were used to analyze the correlation between peripheral blood markers and the onset of irAEs.

**Result:**

NLR < 5, LDH < 240 U/L, or PNI ≥ 45 was favorably associated with significantly better outcomes compared with higher, higher, or lower values, respectively. The multivariate analysis determined that these parameters were independently associated with both better PFS (*p *= 0.049, 0.046, 0.014, respectively) and longer OS (*p *= 0.007, 0.031, < 0.001, respectively). Patients with three favorable factors among NLR, LDH, and PNI had better PFS and OS than did those with two, one, or none. PNI and NLR were associated with the onset of irAEs.

**Conclusion:**

In patients with advanced NSCLC treated with PD-1 inhibitors, pretreatment NLR, LDH, and PNI may be useful predictive markers of clinical outcome and irAEs.

## Introduction

Whether across the globe or in China, lung cancer is the primary cause of cancer-related deaths [1]. Non-small cell lung cancer (NSCLC) accounts for ~ 85% of cases, and a majority include distant metastasis at diagnosis.

In recent years, immunotherapy has been associated with improved long-term survival in advanced NSCLC. The treatment options of patients have vastly expanded, especially through the use immune checkpoint inhibitors [2]. However, only a minority of patients have benefited [3]. There are predictive biomarkers that may be helpful for selecting patients for immunotherapy. Among the most recently recognized are PD-L1 (programmed death-ligand 1), TMB (tumor mutational burden), and MSI-H (microsatellite instability-high). However, these markers are costly, and the testing technology is not yet mature. On the other hand, if peripheral blood markers were known, the test would be clinically convenient and practically noninvasive.

Inflammation is closely linked to cancer, as it promotes a favorable microenvironment for cancer cell growth and spread, and activation of carcinogenic signaling pathways [4]. The prognostic value of some inflammation-related peripheral blood parameters has been investigated, including the neutrophil-to-lymphocyte ratio (NLR), lactate dehydrogenase (LDH), and prognostic nutritional index (PNI). Calculation of the NLR depends on the absolute neutrophil count and the absolute lymphocyte count within the peripheral blood; some studies have shown that NLR is associated with worsened prognosis in patients with melanoma receiving immunotherapy [5–7]. Similarly, a high pretreatment NLR is putatively a poor prognostic indicator in NSCLC [8–10]. So too, high serum LDH is reportedly a risk factor of poor prognosis in patients with NSCLC [11–13].

The PNI is based on serum albumin level and total lymphocyte count. It is easily calculated in daily routine, and a simple means to assess perioperative immunological and nutritional condition and stratify the risk of postoperative complications [14]. The preoperative or pretreatment PNI status is a good prognostic indicator in various cancers, including lung [15, 16], gastric [17], and colorectal [18] cancers, and glioblastoma [19]. However, sufficient data regarding the application of the PNI in the field of cancer immunotherapy are lacking.

Although patients can better tolerate immune checkpoint inhibitors compared with traditional chemotherapy, some patients still suffer adverse events which were known as immune-related adverse events (irAEs) that may cause treatment discontinuation or even death. Markers that may predict the onset of irAEs are unknown.

This retrospective study explored associations between inflammation-related peripheral blood markers (NLR, LDH, and PNI), and outcome and onset of irAEs in patients with advanced NSCLC receiving PD-1 inhibitors.

## Materials and methods

### Patients

The Research Ethics Board of First Affiliated Hospital of Nanchang University approved this retrospective study. The study population comprised 102 patients with a histologically or cytologically proven diagnosis of advanced NSCLC (IIIB/IV), who were treated with anti-PD-1 antibodies at our hospital from January 2017 to May 2019. Further inclusion criteria were an Eastern Cooperative Oncology Group performance status (ECOG PS) of 0–2, and ≥ 4 cycles of immunotherapy treatment. Patients with any of the following were excluded from this study: autoimmune disease; pulmonary interstitial disease; adrenal insufficiency; or systemic immunosuppression.

### Treatment and data collection

Patients received the following, until tumor progression, development of unacceptable drug toxicity, withdrawal, or death: nivolumab and toripalimab (intravenously 3 mg/kg every 2 weeks); sintilimab (200 mg every 3 weeks); and pembrolizumab (2 mg/kg every 3 weeks). The patients’ clinical characteristics were collected, including age, gender, histology, sensitive gene mutation status and so on.

Baseline measurements were defined as those taken within 1 week before receiving PD-1 inhibitors. The baseline peripheral blood data including absolute neutrophil count, LDH, absolute lymphocyte count, total lymphocyte count, and serum albumin level were mostly used to compute the NLR (absolute neutrophil count divided by absolute lymphocyte count) and the PNI (10 × serum albumin value, g/dL + 0.005 × total lymphocyte count/mm^3^).

### Study assessments

Drug efficacy was assessed every 8–12 weeks based on the Response Evaluation Criteria in Solid Tumors (RECIST, version 1.1) by computed tomography (CT) scan. The data deadline was August 2019. The Common Terminology Criteria for Adverse Events (CTCAE) of the National Cancer Institute (version 4.03) was used to assess patients’ adverse events. The irAEs were defined as adverse events that reflected a disorder of the immune system, such as rash, colitis, liver dysfunction, thyroid disorder, and other conditions. The irAEs were evaluated for 3 months, because the incidence of irAEs has been reported to be highest within the first 12 weeks [20].

Progression-free survival (PFS) was defined as the first day of immunotherapy to the date of disease progression. Overall survival (OS) was from the time of initial anti-PD-1 immunotherapy to death from any cause or last follow-up.

### Statistical analysis

Patients were stratified as low- and high-NLR (< 5 and ≥ 5, respectively) [10], low- and high-LDH (< 240 and ≥ 240 U/L) [12], or low- and high-PNI (< 45 and ≥ 45) groups [21]. These cutoffs were selected according to literature references 10, 12, 21 and their median, respectively.

PFS and OS curves were calculated using the Kaplan–Meier method, and the log-rank test was employed to assess differences. Cox regression models were applied to find independent indicators associated with PFS and OS. Factors which were statistically significant in the univariate analysis were incorporated into the multivariate analysis. Logistic regression analysis was applied to explore the correlation between peripheral blood markers and the onset of irAEs. SPSS 25.0 software (*SPSS*, *Chicago, IL*) was used for all the statistical tests. A *p* value< 0.05 was considered statistically significant.

## Results

### Patient characteristics

In our study, 102 patients were enrolled who accepted at least four cycles of immunotherapy (Table 1). Every patient was administered monotherapy with PD-1 inhibitor; 19 patients accepted PD-1 inhibitors as first-line treatment. The median age was 62 years. Most were men (87/102, 85.3%); most had no or undetected sensitive gene mutations (94/102, 92.2%); and most had an ECOG performance status of 0–1 (89/102, 87.3%).

**Table 1:** Patient characteristics

Feature	N	Percentage (%)
Gender	102	100
Female	15	14.7
Male	87	85.3
Age (years)		
< 60	39	38.2
≥ 60	63	61.8
Histology		
Adenocarcinoma	58	56.9
Squamous carcinoma	42	41.2
Large cell carcinoma	2	1.9
Clinical stage		
IIIB	40	39.2
IV	62	60.8
ECOG PS		
0–1	89	87.3
2	13	12.7
Smoking status		
Never	41	40.2
Former/current	61	59.8
Line of immunotherapy		
First	19	18.6
Second	51	50.0
≥ Third	32	31.4
Actionable mutation		
(–)/undetected	94	92.2
EGFR or ALK/ROS1(+)	8	7.8
Number of metastatic sites		
< 3	46	45.1
≥ 3	56	54.9
Immune adverse events		
No	63	61.8
Yes	39	38.2
Type of immunotherapy		
Nivolumab	11	10.8
Pembrolizumab	26	25.5
Toripalimab	30	29.4
Sintilimab	35	34.3

*ECOG PS* Eastern Cooperative Oncology Group performance status, *EGFR* epidermal growth factor receptor, *ALK* anaplastic lymphoma kinase, *ROS1* c-ros oncogene 1

### Univariate and multivariate analyses of biomarkers for OS and PFS

**Fig. 1:**
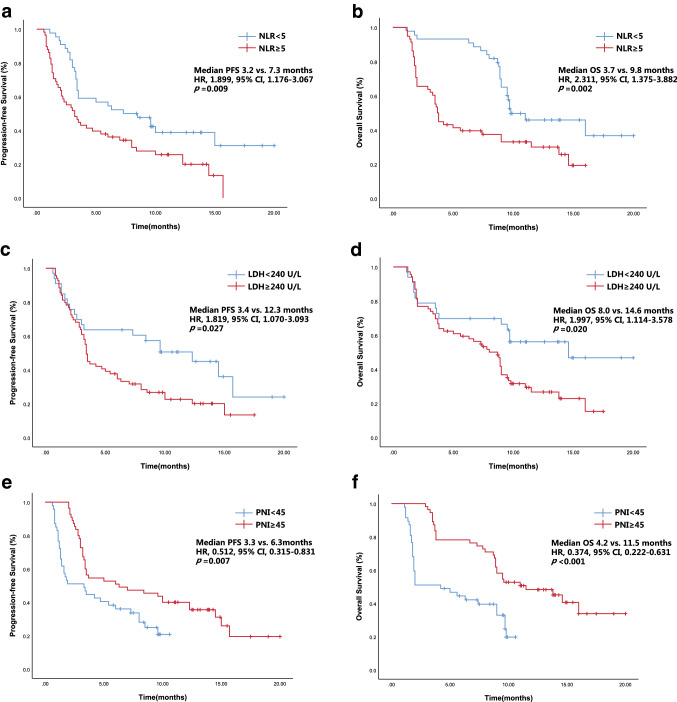
PFS (**a**, **c**, **e**) and OS (**b**, **d**, **f**) curves of patients stratified according to peripheral blood markers (NLR, LDH, and PNI)

For the population overall, the median OS and PFS were 9 months and 3.7 months, respectively. According to the univariate analysis, the high-NLR group had a significantly worse median OS (3.7 months) and median PFS (3.2 months) compared with the low-NLR group (9.8 months and 7.3 months, respectively; Table 2). The high-LDH group had a significantly worse median OS (8.0 months) and median PFS (3.4 months) compared with the low-NLR group (14.6 months and 12.3 months). The high-PNI group had a significantly better median OS (11.5 months) and median PFS (6.3 months) compared with the low-PNI group (4.2 months and 3.3 months). The multivariate analysis showed that the following factors were significantly associated with OS and PFS (Table 2): NLR ≥ 5, LDH ≥ 240 U/L, and PNI ≥ 45 (Fig. 1).

**Table 2:** Univariate and multivariate analyses of PFS and OS

Variable	Category	PFS						OS					
		Univariate			Multivariate			Univariate			Multivariate		
		HR	95%CI	*p* value	HR	95%CI	*p* value	HR	95%CI	*p* value	HR	95%CI	*p* value
Gender	Female	0.780	0.418–1.457	0.436				0.945	0.493-1.810	0.865			
Age	≥ 60	1.585	0.970–2.590	0.066				1.581	0.936–2.671	0.087			
Histology	Non-adenocarcinoma	0.999	0.792–1.262	0.997				0.929	0.726–1.190	0.562			
Clinical stage	IV	0.916	0.723–1.160	0.468				0.916	0.710–1.180	0.497			
ECOG PS	2	1.503	0.769–2.936	0.233				1.496	0.738–3.036	0.264			
Smoking status	Former/Current	1.231	0.977–1.551	0.078				1.240	0.967–1.589	0.090			
Line of immunotherapy	≥3	0.957	0.751–1.220	0.722				0.970	0.747–1.261	0.822			
Actionable mutations	Undetected/(–)	0.712	0.481–1.055	0.090				0.713	0.480–1.059	0.093			
Metastatic sites	≥ 3	1.021	0.810–1.285	0.863				1.008	0.788–1.291	0.947			
Adverse events	Yes	1.344	1.051–1.717	**0.018**	1.011	0.730–1.402	0.945	1.426	1.096–1.855	**0.008**	0.957	0.675–1.356	0.804
NLR	≥ 5	1.899	1.176–3.067	**0.009**	1.845	1.002–3.399	**0.049**	2.311	1.375–3.882	**0.002**	2.491	1.288–4.819	**0.007**
PNI	≥ 45	0.512	0.315–0.831	**0.007**	0.520	0.308–0.877	**0.014**	0.374	0.222–0.631	**<0.001**	0.358	0.202–0.636	**<0.001**
LDH	≥ 240	1.819	1.070–3.093	**0.027**	1.730	1.010–2.963	**0.046**	1.997	1.114–3.578	**0.020**	1.922	1.061–3.481	**0.031**

*PFS* progression-free survival, *HR* hazard ratio, *CI* confidence interval, *ECOG PS* Eastern Cooperative Oncology Group performance status, *NLR* neutrophil-to-lymphocyte ratio, *PNI* prognostic nutrition index, *LDH* lactate dehydrogenase

Statistically significant values are in bold (*p *< 0.05)

### Multifactor model for survival outcome of patients treated with PD-1 inhibitors

**Fig. 2:**
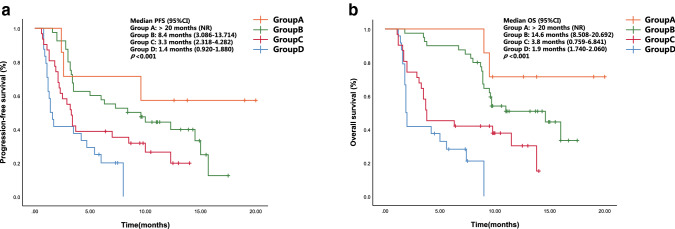
PFS (**a**) and OS (**b**) curves of the multifactor model according to the number of advantageous factors at baseline (NLR < 5, LDH < 240 U/L, and PNI ≥ 45). Abbreviation: NR, not reached

We explored the OS and PFS by the number of advantaged factors (i.e., NLR < 5, LDH < 240 U/L, and PNI ≥ 45; Fig. 2). Of the 102 patients, 24 (23.5%) had no favorable factors (group D, in Fig. 2). The OS and PFS of this group were significantly shorter than that of patients with 1, 2, or 3 of the favorable factors (groups C, B, and A; *p* < 0.001).

### Immune-related adverse events (irAEs)

**Fig. 3:**
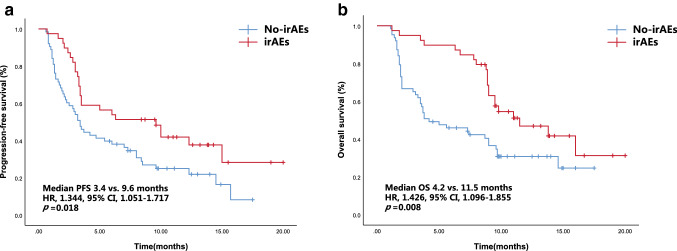
PFS (**a**) and OS (**b**) curves of patients according to the onset of irAEs

Thirty-nine patients (38.2%) experienced, in all, 6 different irAEs of any grade, and 6 patients (5.8%) experienced high-grade irAEs. The commonly seen irAEs (any grade) were rash (*n* = 13, 33.3%), liver dysfunctions (*n* = 9, 23.1%), and hypothyroidism (*n* = 7, 17.9%). The most common severe irAEs (grade ≥ 3) were rash (*n* = 2, 5.1%). The median OS of the 39 patients with irAEs (11.5 months) was significantly preferable to that of the 63 patients without irAEs (4.2 months; Table 2, Fig. 3). Similarly, the median PFS of the patients with irAEs (9.6 months) was significantly better than that of patients without irAEs (3.4 months). However, irAEs were not an independent prognostic risk factor of OS and PFS in the multivariate analysis.

The low-NLR, low-LDH, and high-PNI groups consisted of 32 (72.7%), 15 (45.5%), and 30 (54.5%) patients, respectively (Table 3). The univariate analysis indicated associations between low-NLR or high-PNI and any grade of irAEs (*p* < 0.001, both). The multivariate logistic regression analysis showed that high PNI (*p* = 0.001) and low NLR (*p* < 0.001) were independent predictors for the onset of irAEs.

**Table 3:** Levels of the peripheral blood markers by irAE development

Blood parameter	irAEs, *n* (%)	Univariate		Multivariate	
		OR (95%CI)	*p* value	OR (95%CI)	*p* value
L-NLR (*n* = 44)	32 (72.7)	0.051(0.02–0.14)	**<0.001**	0.04 (0.01–0.13)	**<0.001**
H-NLR (*n* = 58)	7 (12.1)	1		1	
L-LDH (*n* = 33)	15 (45.5%)	0.64(0.28–1.49)	0.301	0.45 (0.14–1.41)	0.169
H-LDH (*n* = 69)	24 (34.8%)	1		1	
H-PNI (*n* = 55)	30 (54.5%)	5.07(2.06–12.4)	**<0.001**	7.61 (2.20–26.3)	**0.001**
L-PNI (*n* = 47)	9 (19.1%)	1		1	

*OR* odds ratio, *CI* confidence interval, *H-NLR* high NLR, *H-LDH* high LDH, *H-PNI* high PNI, *irAEs* immune-related adverse events, *NLR* neutrophil-to-lymphocyte ratio, *LDH* lactate dehydrogenase, *PNI* prognostic nutrition index, *L-NLR* low NLR, *L-LDH* low LDH, *L-PNI* low PNI

## Discussion

Although the preciseness of lung cancer treatment has improved significantly in recent years, NSCLC remains challenging. The emergence of PD-1 inhibitors has brought hope to patients with advanced NSCLC, but many clinical studies have shown that no more than 20% of patients benefit. Therefore, effective predictive biomarkers are urgently needed for screening potential beneficial groups.

PD-L1 is highly expressed on the cell membranes of NSCLC. Anti-PD-1 immunotherapy of NSCLC is designed to block the signal between PD-1 on T cells and PD-L1 on tumor cells [22]. Graves et al. [23] reported that the PD-1 level on CD4+ T cells in the blood of melanoma patients who responded to anti-PD-1 therapy was higher than that of non-responders. Currently, the PD-L1 level is a commonly used marker for predicting the efficacy of immunotherapy. As reported by CheckMate-057 [24] and Keynote-010 [25], patients with high PD-L1 levels in tumor tissues, and who received PD-1/PD-L1 inhibitors, had better survival outcomes compared with those who were not given this treatment. Nevertheless, CheckMate-017 [26] reported that patients who were PD-L1-negative also responded well. Therefore, PD-L1 level is not sufficient as the sole decisive predictor of immunotherapy. TMB is another potential predictive biomarker that has received much attention, but has been considered only as a reference marker; TMB should be explored further in clinical research. In May 2017, pembrolizumab received approval by the United States Food and Drug Association for the treatment of metastatic or advanced solid tumors with mismatch repair deficiency (i.e., high levels of microsatellite instability, or MSI-H). However, the American Society of Clinical Oncology (ASCO) reported in 2016 that MSI-H occurs in only 0.4–0.8% of lung cancer. The predictive markers discussed above are limited by cumbersome detection protocols and high cost. Hence, it is necessary to explore for markers that can effectively predict the benefit of therapy, but which are also clinically practical and without serious drug toxicity.

It has been reported that nutritional status and inflammatory status have prognostic relevance in patients with a variety of cancers [27, 28]. The markers evaluated in the present study (NLR, LDH, and PNI) reflect well the inflammation and nutritional status. The association between baseline NLR and the prognosis of melanoma patients treated with immune checkpoint inhibitors has been demonstrated [29, 30]. Bagley et al. [10] studied 175 patients with advanced NSCLC treated with nivolumab and concluded that NLR ≥ 5 at baseline was a risk factor of inferior OS (HR 1.83, 95% CI 1.2–2.8; *p* = 0.006) and inferior PFS (HR 1.42, 95% CI 1.02–2.0; *p* = 0.04), compared with NLR < 5. In the multivariate analysis, NLR ≥ 5 was also independently linked to worse outcomes. In addition, another retrospective study showed that baseline NLR > 5 was associated with poor OS [31]. In the present study, we also concluded that an NLR of 5 was the optimal cutoff: NLR ≥ 5 was associated with worse OS (HR 2.311, 95% CI 1.375–3.882; *p* = 0.002) and PFS (HR 1.899, 95% CI 1.176–3.067; *p* = 0.009). Furthermore, NLR ≥ 5 was an independent prognostic risk indictor in the multivariate analysis model.

LDH is produced by rapidly growing tumors and therefore reflects the tumor burden. LDH was found associated with outcomes in melanoma patients treated with immune checkpoint inhibitors [32, 33]. Several studies used LDH to forecast PFS [12, 34] and OS [6, 11] in patients with NSCLC treated with immune checkpoint inhibitors. Taniguchi et al. [12] discovered that among patients with advanced NSCLC treated with nivolumab, those with baseline LDH > 240 U/L had a significantly worse PFS compared with those with LDH ≤ 240 U/L. Similarly, the present analysis showed that a baseline LDH ≥ 240 U/L was associated with worse OS and PFS.

In 1984, Japanese scholars Onodera et al. [35] proposed a simplified version of the PNI, which is based only on serum albumin and total the lymphocyte count. Serum albumin concentration can indicate the nutritional status of patients and chronic inflammation. In addition to reflected inflammatory status, the lymphocyte count is also an important parameter of the immune function [36]. Several studies have reported a significant association between the PNI and the prognosis of patients with a variety of malignant cancers. However, there has been little research regarding the value of the PNI in cancer immunotherapy. In the present study, a pretreatment PNI ≥ 45 was associated with better OS (HR 0.374, 95% CI 0.222–0.631; *p* < 0.001) and PFS (HR 0.512, 95% CI 0.315–0.831; *p* = 0.007) compared with PNI < 45. In the multivariate analysis, PNI ≥ 45 was also independently associated with worse PFS and OS. An evaluation of baseline PNI may provide meaningful information for selecting suitable patients in immunotherapy.

The increasing use of immune checkpoint inhibitors has been accompanied by a rise in unique adverse events, known as irAEs, which can lead to troubling morbidity and treatment discontinuations [37]. Nakaya et al. [38] reported that patients with NSCLC treated with nivolumab and who experienced an associated irAEs had better PFS compared with those who had no irAEs. Similarly, the present analysis showed that the patients who suffered from irAEs had longer PFS and OS, although this did not rise to the level of an independent prognosis marker for PFS and OS. Moreover, the rate of irAEs in our research also resembled the study that reported by Nakaya et al. [38]. The current study also explored an association between irAEs and the peripheral blood markers NLR, LDH, and PNI and found that low NLR and high PNI were significantly associated with the onset of irAEs. So, the baseline NLR and PNI may be used as a convenient tool to identify irAEs timely, which is essential for reducing the risk of hospitalization and the costs of treatment.

The limitations of this research are its retrospective nature and relatively small sample size. Although the results of our study are interesting, the predictive value of the peripheral blood markers (NLR, LDH, and PNI) on PFS, OS or irAEs requires further validation by randomized studies with an untreated control group. Furthermore, most of the patients received PD-1 inhibitors as their second-line treatment or beyond. Thus, the degree of baseline inflammation may be affected by previous treatments, although the number of lines of immunotherapy was not associated with clinical outcomes. Additionally, the PD-L1 status was known in so few patients that we could not include in our study and was conducted using different methods. Finally, immune-related response criteria were not applied in this study, because it was not a prospective research and most physicians are unfamiliar with the criteria. Despite these limitations, a unique aspect of this study was the combined model of three baseline peripheral blood markers for the outcome of PD-1 inhibitors. In addition, to our best knowledge, it is the first study to explore a correlation between peripheral blood markers (LDH and PNI) and irAEs in patients with advanced NSCLC accepting PD-1 inhibitors, or an evaluation of the effect of irAEs on both PFS and OS.

In conclusion, the pretreatment peripheral blood markers analyzed herein (NLR, LDH, and PNI) may correlate with outcomes and the onset of irAEs in patients with advanced NSCLC accepting PD-1 inhibitors. This provides some directions for clinical research on immunotherapy of lung cancer. With the rising attention to health-related costs and precision medicine, the predictive role of peripheral blood markers in cancer immunotherapy is very meaningful. These preliminary results warrant further research.

## Abbreviations

AE: Adverse event
ALK: Anaplastic lymphoma kinase
CI: Confidence interval
CT: Computed tomography
ECOG PS: Eastern Cooperative Oncology Group performance status
EGFR: Epidermal growth factor receptor
HR: Hazard ratio
irAEs: Immune-related adverse events
LDH: Lactate dehydrogenase
MSI-H: Microsatellite instability-high
NLR: Neutrophil-to-lymphocyte ratio
NR: Not reached
NSCLC: Non-small cell lung cancer
OR: Odds ratio
OS: Overall survival
PD-1: Programmed cell death-1
PD-L1: Programmed death-ligand 1
PFS: Progression-free survival
PNI: Prognostic nutrition index
ROS1: c-ros oncogene 1
TMB: Tumor mutational burden
